# Multilevel pharmacokinetics-driven modeling of metabolomics data

**DOI:** 10.1007/s11306-017-1164-4

**Published:** 2017-02-08

**Authors:** Emilia Daghir-Wojtkowiak, Paweł Wiczling, Małgorzata Waszczuk-Jankowska, Roman Kaliszan, Michał Jan Markuszewski

**Affiliations:** Department of Biopharmaceutics and Pharmacodynamics, Medical University of Gdańsk, Al. Gen. Hallera 107, 80-416 Gdańsk, Poland

**Keywords:** Multi-level modeling, Bayesian analysis, Pharmacokinetics, Metabolomics, Nucleosides, Methylthioadenosine, Cancer

## Abstract

**Introduction:**

Multilevel modeling is a quantitative statistical method to investigate variability and relationships between variables of interest, taking into account population structure and dependencies. It can be used for prediction, data reduction and causal inference from experiments and observational studies allowing for more efficient elucidation of knowledge.

**Objectives:**

In this study we introduced the concept of multilevel pharmacokinetics (PK)-driven modelling for large-sample, unbalanced and unadjusted metabolomics data comprising nucleoside and creatinine concentration measurements in urine of healthy and cancer patients.

**Methods:**

A Bayesian multilevel model was proposed to describe the nucleoside and creatinine concentration ratio considering age, sex and health status as covariates. The predictive performance of the proposed model was summarized via area under the ROC, sensitivity and specificity using external validation.

**Results:**

Cancer was associated with an increase in methylthioadenosine/creatinine excretion rate by a factor of 1.42 (1.09–2.03) which constituted the highest increase among all nucleosides. Age influenced nucleosides/creatinine excretion rates for all nucleosides in the same direction which was likely caused by a decrease in creatinine clearance with age. There was a small evidence of sex-related differences for methylthioadenosine. The individual *a posteriori* prediction of patient classification as area under the ROC with 5th and 95th percentile was 0.57(0.5–0.67) with sensitivity and specificity of 0.59(0.42–0.76) and 0.57(0.45–0.7), respectively suggesting limited usefulness of 13 nucleosides/creatinine urine concentration measurements in predicting disease in this population.

**Conclusion:**

Bayesian multilevel pharmacokinetics-driven modeling in metabolomics may be useful in understanding the data and may constitute a new tool for searching towards potential candidates of disease indicators.

**Electronic supplementary material:**

The online version of this article (doi:10.1007/s11306-017-1164-4) contains supplementary material, which is available to authorized users.

## Introduction

Multilevel (hierarchical) modeling, also known as random-effects or mixed-effects modeling, is a generalization of regression methods known to outperform classical regression in predictive accuracy. This approach is becoming increasingly popular and the general motivation to use it lies in (i) adjusting estimates in case of repeated sampling (e.g. in time-series cross-sectional data) (ii) adjusting estimates in case of imbalance in sampling (e.g. when unbalanceness occurs in the data) (iii) modeling variation among individuals or groups in the data, (iiii) regularization of parameter estimates with large number of predictors (McElreath 2014; Gelman and Feller 2015).

The concept of multilevel modeling constitutes a domain in pharmacokinetics (PK) for time-series data (Rodrigues et al. 2013) and has recently been established in the field of nutrikinetics (NK) (van Velzen et al. 2009).

Pharmacokinetics (PK) is a study of the time course of absorption, distribution, metabolism and excretion (ADME) of a drug and how these processes vary between individuals. In its general form, PK-based modeling provides mechanistic characterization of the observed concentration–time profile of a drug in order to gain more insights towards understanding the phenomena associated with drug absorption and disposition (Liu et al. 2013; Bonate et al. 2005). In PK, multilevel modeling is an often used tool to describe relationship between PK parameter and individual characteristics (e.g. age, sex, weight, smoking status, renal function) in terms of fixed effects (associated with covariates) and random effects (unexplained variation between individuals) (Gelman et al. 1996; Imlach Gunasekara et al. 2014). In principle, it shares similarities to metabolomics with the difference only related to the number of compounds analyzed at a time.

Nutrikinetics (NK) in turn, is an application area of pharmacokinetics which studies the ADME of food compounds or dietary supplements in the human organism including the interaction within the host metabolome and the gut microbiome (van Velzen et al. 2008). In NK, multilevel partial least squared discriminant analysis (PLS-DA) models are used to account for between-subject variability and intra-individual variability (IIV) in order to develop classification models for longitudinal studies (Westerhuis et al. 2010).

Compared to NK, the aim of multilevel PK-driven modeling does not lie in classification, but is mainly focused on modeling the concentration in a function of many different-type covariates (numerical, categorical, binary). Such an approach allows for characterization of biological or physiological phenomena in the system observed for each individual simultaneously accounting for variability (Lavielle 2014). From PK viewpoint, excretion into urine is one of the route of elimination of drugs and low molecular weight metabolites, and its rate can be predicted from creatinine clearance. From PK viewpoint, endogenous creatinine clearance or its indirect estimation is generally used to establish drug dosage for urinary excreted drugs. In metabolomics, creatinine is an indicator of urine dilution widely used for the purpose of normalization of metabolites determined in urine (Lindon et al. 2007).

Creatinine clearance can be estimated via the Cockcroft-Gault formula from age, sex, weight and serum creatinine or from the Modification of Diet in Renal Disease Study (MDRD) which considers age, sex and serum creatinine. Therefore, we can expect that any differences in terms of age, sex or weight between individuals affect the production rate of creatinine between individuals (Levey et al. 1999; Cockcroft et al. 1976). This phenomenon has a large impact in metabolomics observational studies for those metabolites that are determined in urine as their concentration is usually normalized by creatinine clearance. In such a case, using traditional multivariate methods, e.g. PLS-DA, to discriminate between groups investigated, might lead to improper conclusions as any imbalance in age or sex will lead to biased inference of group difference.

Multilevel modeling has been used in unbalanced study design involving longitudinal studies covering individuals characterized by a wide age range (Morrell et al. 2009). However, the applicability of multilevel modeling can also be extended to cross-sectional studies (at one specific time point) within nested and non-nested scenario (Gelman et al. 2007; Gelman 2006). In this context, the idea of multilevel modeling fits into metabolomics studies which often suffer from small data sets, high uncertainty and biological variability, making data modeling difficult (Bernillon et al. 2000). So far, in the field of metabolomics little attention has been paid to develop or propose a modeling strategy that would account for the problem related to variability or uncertainty.

In this work we introduce the concept of multilevel PK-driven modeling within the Bayesian framework as a method to cope with large-sample, unbalanced, sex and age-unadjusted data. In this study, metabolomics data involving measurement of 13 nucleosides in urine of healthy and urogenital track cancer patients with corresponding age and sex, were considered. Multilevel PK-driven regression model within Bayesian framework was used by assuming probability distribution for all model parameters. The model developed assumed normally-distributed regularizing prior for case/control effect and informative priors form the MDRD formula imposed on age and sex. Accordingly, the model proposed was characterized via relationships between patient characteristics (age, sex, case/control status) and model parameters. Accordingly, we evaluated the posterior probability of cancer occurrence in an individual person along with providing the classification performance of the model via area under the ROC, sensitivity and specificity. At the end we discuss the pros and cons of traditional hypothesis testing strategy versus multilevel PK-driven modeling in the context of metabolomics.

## Methods

### Data set used in the study

The data set used in this study consisted of *n* = 248 individuals (153 patients and 95 healthy individuals) and *p* = 13 nucleosides concentrations determined using RP-HPLC with UV–Vis spectrophotometric detection previously published by Waszczuk-Jankowska et al. (2012) in terms of the utility of different stationary phases for the determination of 13 nucleosides as potential cancer biomarkers. In the above-mentioned paper, concentrations of 13 metabolites normalized by creatinine clearance were compared between healthy and urogenital tract cancer patients using Mann–Whitney U test statistics for all study participants. Significant differences were observed in 10 out of 13 nucleosides. More detailed description of the study participants, experimental conditions (LC instrumentation and chromatographic conditions, stationary phases selection) and urine sample collection procedure, can be found therein.

In the present study, the same data set of 13 nucleosides’ concentrations with additional covariates such as age and sex were considered and subjected to multilevel PK-driven data analysis.

### Model development

Dataset used in this study consisted of standardized and mean-centered *n*×*p* matrix (Y) representing the natural logarithm of nucleosides to creatinine concentration ratios. The dataset Y was randomly divided into training (70%) and validation (30%) set, Y = (Y_train_, Y_val_). Age, sex, and health status constituted the available covariates, where X = (X_train_, X_val_) denotes the *n*×2 matrix covariate with age and sex as column vectors and Z = (Z_test_, Z_val_) denotes *n*×1 vector describing health status (cancer versus healthy). The *Z*_val_ values were further treated as missing to assess the predictive performance of the model in identifying disease status of an individual subject.

Assuming steady-state conditions, the measured urine concentrations of nucleoside *h* (*h* = 1…*p*) in individual *i* (*i* = 1...*n*) expressed as *m*_ih_ is given by:

1 $$\ln {{m}_{\text{ih}}}=\ln \frac{k{}_{\text{m,ih}}}{{{V}_{\text{0,i}}}}+{{\varepsilon }_{\text{m,ih}}}$$

Similarly, measured concentration of creatinine in individual *i* (*c*_i_) is given by:

2 $$\ln {{c}_{\text{i}}}=\ln \frac{k{}_{\text{c,i}}}{{{V}_{\text{0,i}}}}+{{\varepsilon }_{\text{c,i}}}$$

where $k{}_{\text{m,ih}}$ is the excretion rate (the product of clearance and unbound plasma concentration) of nucleoside *h* and $k{}_{\text{c,i}}$ is the excretion rate of creatinine in individual *i;* ${{V}_{\text{0,i}}}$ is the individual rate of diuresis (assumed to be equal for all metabolites and creatinine in individual *i*). The model was parameterized in terms of the natural log of the parameter values [e.g. ln(*k*_m,zi_)] to simulate the usual lognormal distribution of concentration measurement in biology. The ${{\varepsilon }_{\text{m,ih}}}$ and ${{\varepsilon }_{\text{c,i}}}$ represent an additive (on a log scale) random error for concentration measurements. It was assumed that *ε* is normally distributed with mean 0 and variance–covariance denoted by ${{\Sigma }_{\text{m}}}$ and variance $\sigma _{\text{c}}^{2}$ for nucleosides and creatinine, respectively.

The normalization of nucleosides concentration data by creatinine leads to diuresis-independent relationship:

3 $$\ln \frac{{{m}_{\text{ih}}}}{{{c}_{\text{i}}}}=\ln \frac{k{}_{\text{m,ih}}}{k{}_{\text{c,i}}}+{{\varepsilon }_{\text{m,ih}}}-{{\varepsilon }_{\text{c,i}}}$$

Inter-individual variability (IIV) for all underlying parameter (*k*_m_ and *k*_c_) is further assumed to follow a lognormal distribution:

4 $$\ln {{k}_{\text{m,ih}}}=\ln {{\beta }_{\text{km,h}}}+{{\eta }_{\text{km,ih}}}$$

5 $$\ln {{k}_{\text{c,i}}}=\ln {{\beta }_{\text{kc}}}+{{\eta }_{\text{kc,i}}}$$

where ${{\beta }_{\text{km,h}}}$ and ${{\beta }_{\text{kc}}}$ is the typical value of this parameter in the population, whereas ${{\eta }_{\text{km,ih}}}$ and ${{\eta }_{\text{kc,i}}}$ are random effects for that parameter with mean 0, variance–covariance ${{\Omega }_{\text{km}}}$ and variance $\omega _{\text{kc}}^{2}$.

Combining both equations yields:

6 $$\ln \frac{{{m}_{\text{ih}}}}{{{c}_{\text{i}}}}=\ln \frac{{{\beta }_{\text{km,h}}}}{{{\beta }_{\text{kc}}}}+{{\varepsilon }_{\text{m,ih}}}-{{\varepsilon }_{\text{c,i}}}+{{\eta }_{\text{m,ih}}}-{{\eta }_{\text{c,i}}}$$

It can be compactly expressed as:

7 $${{y}_{\text{ih}}}={{\beta }_{\text{h}}}+{{\kappa }_{\text{ih}}}$$

where ${{\beta }_{\text{h}}}$ is a population mean of the logarithm of the ratio of nucleosides to creatinine excretion rates and ${{\kappa }_{\text{ih}}}$ is a random variable with mean 0 and variance–covariance denoted by $\Lambda$. As shown in Eq. (6), $\Lambda$ can be decomposed into between-subject variabilities in nucleosides and creatinine excretion rates and their respective residual error variabilities. Equations (6) and (7) underlie which aspects of physiology could affect the measured nucleosides to creatinine excretion rates i.e. it depends on the excretion rate of a particular nucleoside and creatinine, whereas residuals are dependent on the measurement error and inter-individual variability of both nucleosides and creatinine.

### Covariates

The potential effect of available covariates (age, sex and case/control status) on nucleoside/creatinine excretion rate was assessed in this study. Since creatinine excretion rate is among others age and sex-dependent, similar effect was assumed for nucleosides. Therefore, the following linear model was proposed in this work:

8 $${{y}_{\text{ih}}}={{\beta }_{\text{0,h}}}+{{\beta }_{\text{age,h}}}\ln ({{x}_{\text{i1}}}/50)+{{\beta }_{\text{sex,h}}}{{x}_{\text{i2}}}+{{\beta }_{\text{cancer,h}}}{{z}_{\text{i}}}+{{\kappa }_{\text{ih}}}$$

where ${{\beta }_{\text{0,h}}}$ is a population mean of the logarithm of the ratio of nucleosides to creatinine excretion rates for a 50 year-old, healthy man. The ${{\beta }_{\text{age,h}}}$ is the effect of age, ${{\beta }_{\text{sex,h}}}$ is the effect of sex, and ${{\beta }_{\text{cancer,k}}}$ characterizes the effect of cancer presence on standardized, mean-centered log ratio of excretion rates of nucleosides and creatinine. It should be noted that based on this data, without additional prior information it is impossible to elucidate whether a given covariate affects creatinine or nucleosides excretion rate.

### Priors

Following the JAGS parameterization (uncertainty is described as a precision, which is an inverse of variance), the stochastic part of the model can be represented as:

9 $${{y}_{\text{ih}}}\sim{}MVN({{\beta }_{\text{0,h}}}+{{\beta }_{\text{age,h}}}\ln ({{x}_{\text{i1}}}/50)+{{\beta }_{\text{sex,h}}}{{x}_{\text{i2}}}+{{\beta }_{\text{cancer,h}}}{{z}_{\text{i}}},{{\Lambda }^{-1}})$$

where *MNV* is multivariate normal distribution with a vector of expected logarithms of ratios of nucleoside/creatinine production rates.

In the model proposed we considered priors for the parameters related to covariates: (i) normally-distributed regularizing prior with mean 0 and standard deviation from the uniform distribution imposed on case/control effect (10), (ii) normally-distributed informative prior with mean 0.203 and uniformly-distributed standard deviation for age (11), and normally-distributed informative prior with mean 0.293 and uniformly-distributed standard deviation for sex (12). The informative priors were elucidated from the MDRD formula (Cockcroft et al. 1976).

The intercept consisted of the vector of hyperprior population mean parameters equal to zero and precision equal to 0.0001 (13). Between/inter-individual subject variability followed Wishart distribution with covariance Λ_0_ = 0.05 *I*_13_ (*I* – identity matrix) and degrees of freedom (ρ= 13) (14):

10 $${{\beta }_{\text{cancer,h}}}\sim{}N(0\text{, 1/}\sigma _{\text{cancer}}^{2}) \, {\text{for nucleosides}}\,h=1... p$$

11 $${{\beta }_{\text{age,h}}}\sim{}N(0.203\text{, 1/}\sigma _{\text{age}}^{2}) \, {\text{for nucleosides}}\,h=1... p$$

12 $${{\beta }_{\text{sex,h}}}\sim{}N(0.293\text{, 1/}\sigma _{\text{sex}}^{2}) \, {\text{for nucleosides}}\,h=1... p$$

13 $${{\beta }_{\text{0,h}}}\sim{}N(0\text{, 0}\text{.0001}) \, {\text{for nucleosides}}\,h=1... p$$

where:

$${\sigma_{cancer}},\,{\sigma_{age}},\,{\sigma _{sex}}\sim{\text{dunif}}\,(0.001,1000)$$

14 $${{\Lambda }^{-1}}\tilde{\ }Wishart(\rho {{\Lambda }_{0}}\text{,}\rho )$$

### Inference about model parameters

The full joint probability of all parameters was obtained using Bayesian framework. The *a posteriori* distribution (up to the proportionality constant) of parameters $\theta$ is

15 $$p(\theta |Y) \propto p(Y|\theta )p(\theta )$$

where $\theta =(\beta ,\Lambda )$ is a set of all unknown model parameters as defined above, *Y* denotes a set of mean-centered and standardized log nucleoside/creatinine ratio measurements. The conditional distribution of $\theta$ given data (i.e.$p(\theta |Y)$) denotes the posterior distribution. The $p(Y|\theta )$ is the distribution of *Y*, which is a likelihood function when viewed as a function of model parameters, assuming that $\theta$ is known, The $p(\theta )$ is the distribution of $\theta$ without any knowledge on data, referred to as a prior distribution. Equation (15) demonstrates that the posterior distribution of $\theta$ is proportional to the product of the likelihood of $\theta$ given *Y* and the prior distribution of $\theta$.

Similarly *a posteriori* predictions can be obtained:

16 $$p(\hat Y|Y) = \int {p(\hat Y|\theta )p(\theta |Y)d\theta }$$

where $\hat Y$ are nucleoside/creatinine ratios predictions.

### Bayesian inferences for cancer development in individual subject

The practical usefulness of metabolomics measurements lies in the inference about the health status of the patients, i.e. elucidation of the probability of cancer presence given a set of nucleosides and creatinine measurements in urine. This problem can be viewed as an inference on the model parameters with missing covariates and can be written as:

17 $$p(\theta ,{{\theta }_{\text{Z}}},{{Z}_{\text{val}}}|Y,{{Z}_{\text{test}}})\propto p(Y|{{Z}_{\text{test}}},{{Z}_{\text{val}}},\theta )p({{Z}_{\text{test}}},{{Z}_{\text{val}}}|{{\theta }_{\text{Z}}})p(\theta )p({{\theta }_{\text{Z}}})$$

Here the likelihood of Y and Z are treated as independent. ${{\theta }_{\text{Z}}}$ denotes a set of parameters for the model of cancer status. The $p({{Z}_{\text{test}}},{{Z}_{\text{val}}}|{{\theta }_{\text{Z}}})$ was obtained assuming:

18 $${{z}_{\text{i}}}\tilde{\ }dbern({{p}_{\text{i}}})$$

where *p*_*i*_ denotes the individual probability of having cancer (thus ${{\theta }_{Z}}={{p}_{\text{i}}}$) estimated from the data assuming diffuse beta prior:

19 $${{p}_{\text{i}}}\tilde{\ }dbeta(1,1)$$

The *p*_*i*_ value corresponds to the individual probability of having cancer and in this work does not reflect the disease prevalence in the population. However, instead of Eq. (19), one can propose prior probability which is more informative, i.e. reflects disease prevalence and influence of different covariates on the probability of cancer occurrence.

### Assessment via posterior predictive check and area under the ROC

Model performance was assessed by means of *a posteriori* predictive check. In this study, 5th, 50th and 95th percentile were used to summarize the data and model predictions. This graph enables the comparison between confidence intervals obtained from prediction and the observed data. When the corresponding percentile from the observed data falls outside the 90% confidence interval derived from predictions, there is an evidence of model misspecification.

The obtained posterior distribution of model parameters allowed for straightforward determination of patients classification performance as healthy or cancer (with uncertainty) by means of the area under the ROC curve, sensitivity and specificity. We used *colAUC* function from *caTools* package to test the classification performance of a classifier separately for training and validation set.

### Technical

The model was developed using JAGS 4.0.0. with *rjags* (Plummer 2015; Plummer et al. 2006), *runjags* (Denwood 2016) and *coda* (Plummer et al. 2015) packages in R environment (R Core Team 2015). Three MCMC chains of 6000 iterations were simulated. The first 3000 iterations of each chain were discarded and every third sample was retained. Thus 3000 MCMC samples were used for subsequent analyses. Model convergence was assessed by Gelman-Rubin diagnostics available in JAGS. The MCMC chains were assumed to have reached the stationary distribution if Gelman-Rubin values were less than 1.2 for all parameters. Furthermore, the trace history of MCMC samples for all chains were examined visually for all parameters, for which ‘fuzzy caterpillar’ suggests that MCMC chains had reached a stationary distribution. All the codes are available in the Supplementary Materials.

## Results and discussion

### Study design and model assumption

The present study constitutes an extension of the work conducted by Waszczuk-Jankowska et al. (2012). In this study, the whole population of 248 individuals (153 patients and 95 healthy individuals) was randomly divided into training (70%) (n = 178) and validation set (30%) (70 individuals). Detail characterization of both sets can be found in Table 1. Both datasets were unbalanced and mismatched in terms of age and sex and therefore, the population used in this study reflected common problems of study design related to mismatch and unbalanceness. In terms of mismatch, such study design hinders the interpretation of results especially when applying a common Frequentist-based approach related to Mann–Whitney U test statistics and correction for multiplicity (false discovery rate (FDR), Bonferroni correction). Unbalanceness, in turn, affects the results provided by any classification method leading to biased results toward majority class and poor predictive performance of a classifier (Chawla 2005).

**Table 1:** Detail characteristics of training and validation set with a set of corresponding covariates (age, sex, case/control status)

	Training set (*n* = 178)		Validation set (*n* = 70)	
	Patients *(n* = 109)	Healthy (*n* = 69)	Patients (*n* = 44)	Healthy (*n* = 26)
Age	64.04 (±11.89)	37.2 (±15.36)	66.22 (±10.42)	43.69 (±17.08)
Sex				
Male	80 (73.4%)	24 (34.8%)	32 (72.7%)	11 (42.3%)
Female	29 (26.6%)	45 (65.2%)	12 (27.3%)	15 (57.7%)

In this work we developed a Bayesian multilevel PK-driven model with prior information on age and sex elicited from the MDRD formula which estimates glomerular filtration rate based on creatinine and patient characteristics. For the case/control effect, we assumed a normally-distributed regularizing prior in order to provide a trade-off between overfitting and underfitting of the model. The concept of regularization is especially important when large number of predictors are introduced into the model or when we expect similar effects between variables (McElreath 2014).

### Development and diagnostic of the regression model

Regression model with standardized, mean-centered nucleosides/creatinine excretion rates on a log scale as dependent variable was developed in a function of case/control effect, age and sex to assess the influence of cancer controlling for difference in age and sex among patients.

**Fig. 1:**
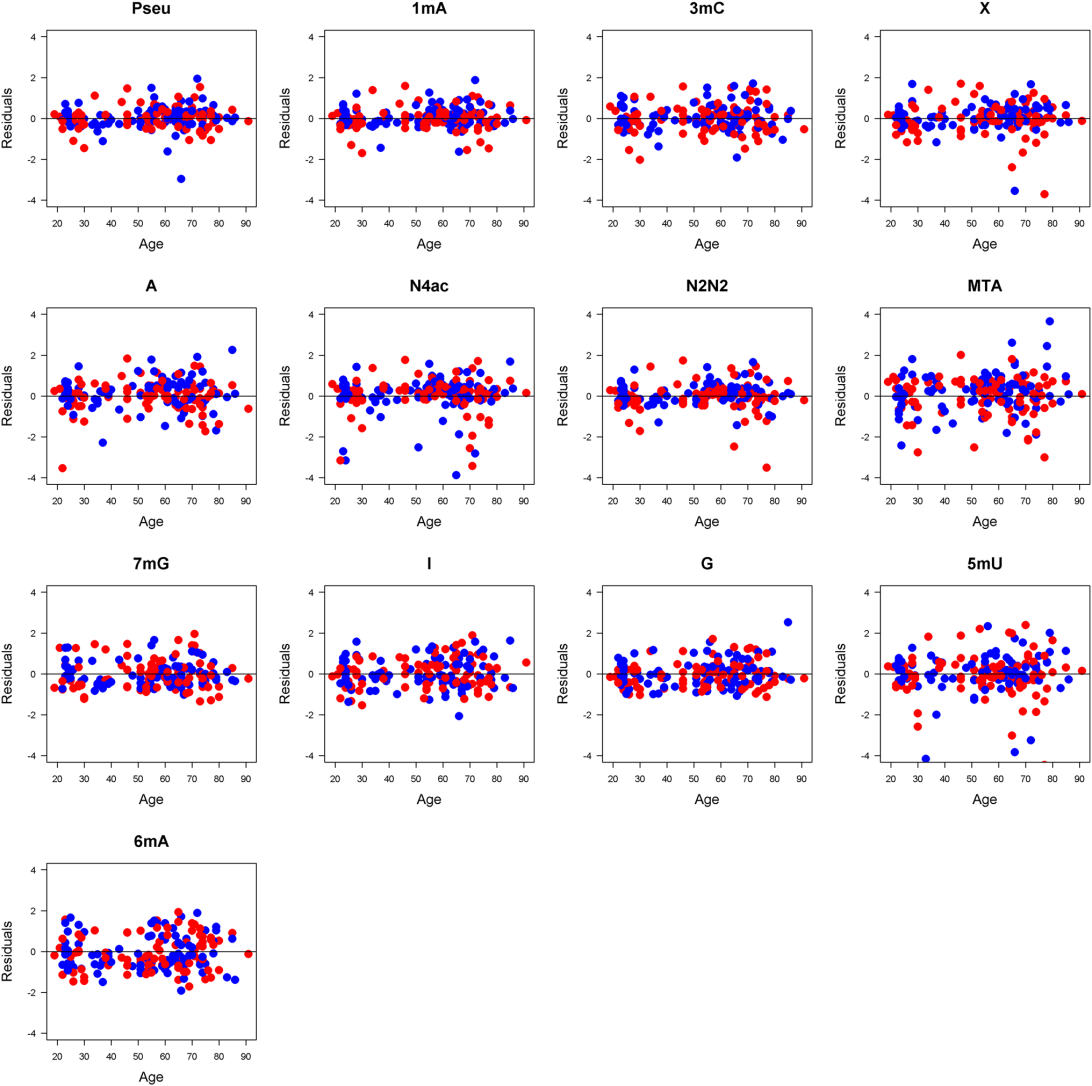
Model residuals in a function of age. *Blue line* represents the regression line, *blue and red dots* represent healthy and cancer patients, respectively. Pseu-pseudouridine; 1mA-1methyadenosine, 3mC-3methylcytidine; X-xanthosine; A-adenosine; N4ac-N4acethylcytidine; N2N2-N2N2dimethylguanosine; MTA-methythioadenosine; 7mG-7methyladenosine; I-inosine; G-guanosine, 5mU-5methyluridine; 6mA-6methyladenosine

Diagnostic of the regression model was performed via checking model residuals and posterior predictive check. No observable pattern in the plot of residuals was reported (no indication of non-constant error variance) therefore no violation from normality assumption can be expected (Fig. 1). We can therefore conclude, that the models of nucleosides/creatinine excretion rates are well fitted in a function of age, sex and case/control effect and thus, no over or under prediction of actual concentration is observed.

**Fig. 2:**
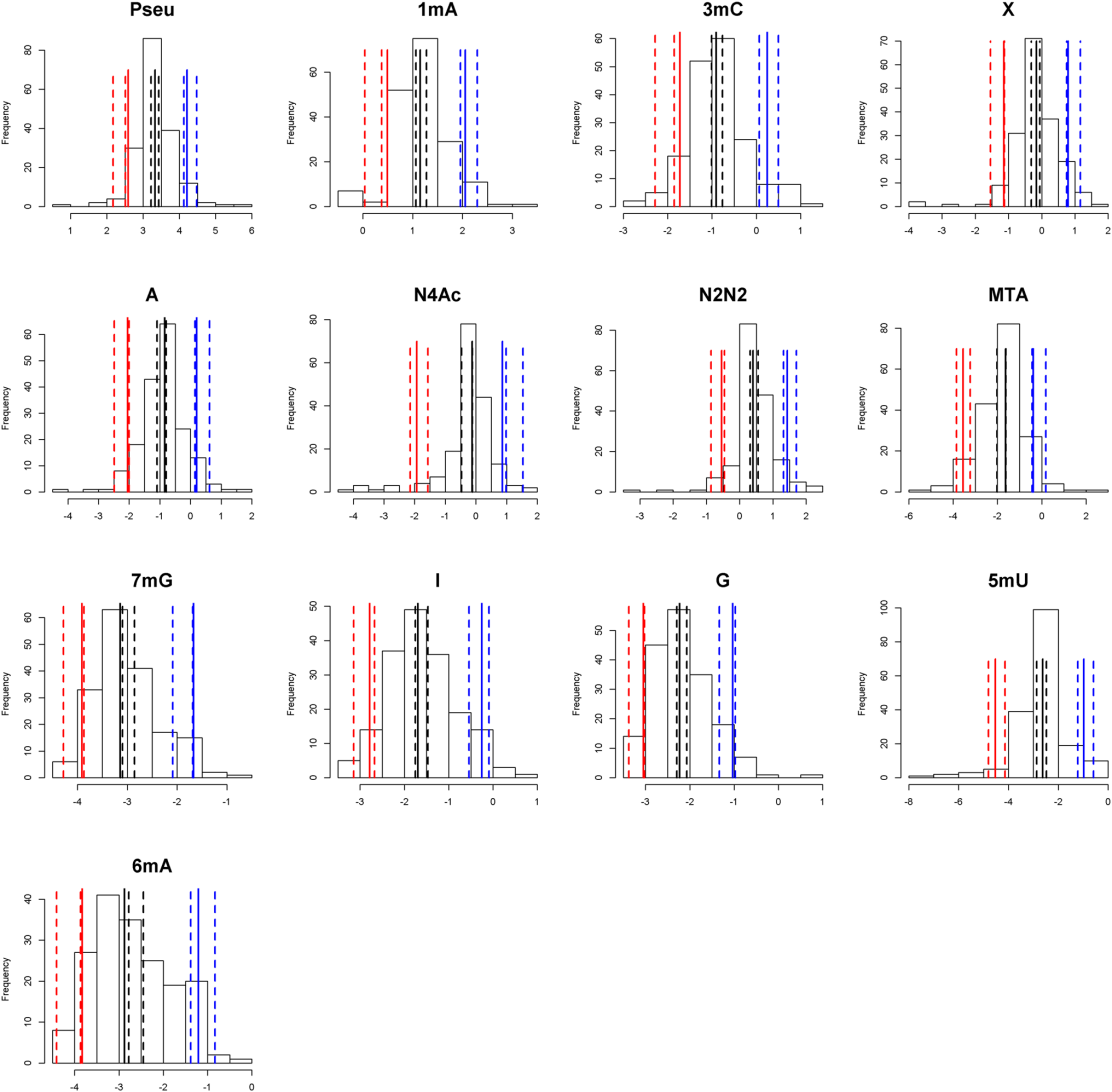
Posterior predictive checks comparing the measured and predicted nucleosides/creatinine concentration ratios. The plot demonstrates 5th (*red*), 50th (*black*) and 95th (*blue*) percentiles for actual (*solid lines*) and posterior data (*dotted lines*). Actual data are presented on histograms

Posterior predictive check compares the actual data to the posterior predictions allowing for making inference whether *a posteriori* predictions from the model are consistent with actual data. Confidence intervals obtained from *a posteriori* predictions and observations were compared as presented in Fig. 2. No systematic differences were observed, therefore we concluded good specification of the model developed on the training set.

**Fig. 3:**
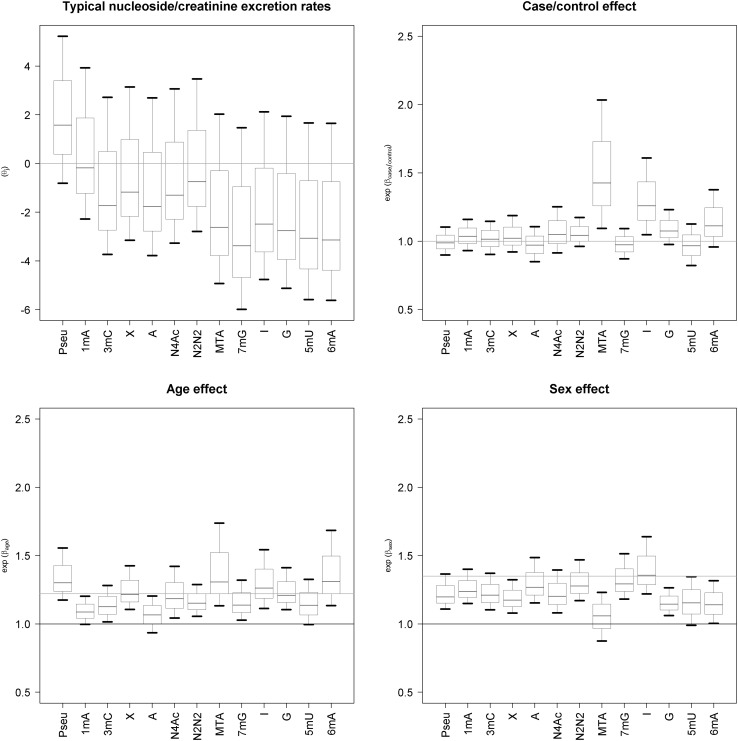
The *a posteriori* distribution of mean ratio of excretion rates of nucleosides to creatinine and the case/control status, age and sex effects on these parameters for each nucleoside tested. The distribution was summarized as a boxplot with 5th, 25th, 50th, 75th and 95th percentile. Pseu-pseudouridine; 1mA-1methyadenosine, 3mC-3methylcytidine; X-xanthosine; A-adenosine; N4ac-N4acethylcytidine; N2N2-N2N2dimethylguanosine; MTA-methythioadenosine; 7mG-7methyladenosine; I-inosine; G-guanosine, 5mU-5methyluridine; 6mA-6methyladenosine

In Fig. 3, we present the typical nucleosides/creatinine excretion rates effect on the case/control status age and sex. As previously observed, as a consequence of cancer, normalized methylthioadenosine (MTA) concentration increased by a factor of 1.42 (1.09–2.03) whereas inosine (I) increased by 1.25 (1.04–1.6) on average, as evidenced by 5th and 95th percentile (assuming the model and the available data). For other nucleosides the mean effect is close to 1 (no effect). Age influences nucleosides/creatinine excretion rates for all nucleosides in the same direction, which is likely caused by a decrease in creatinine clearance with age as can be observed from the MDRD formula (Imai et al. 2007):

20 $${{k}_{\text{c}}}=GFR\cdot {{C}_{\text{Cr}}}\approx 186\cdot Ag{{e}^{-0.203}}\cdot [0.742\ if\ Female]$$

where *k*_c_ denotes creatinine excretion rate, GFR is a glomerular filtration rate and *C*_cr_ indicates plasma creatinine concentration. This equation shows that increase in nucleoside/creatinine excretion ratio by a factor of exp(0.203) = 1.22 should generally be expected due to dependence of creatinine excretion rate on age. Thus our measurements provide an evidence for age-dependent excretion rate for nucleosides as posterior probabilities for age effects (Fig. 3).

The effect of sex is different for different nucleosides/creatinine excretion rates. The value of nucleosides/creatinine excretion rates for pseu, 1 mA, 3mC, X, A, N4Ac, N2N2, 7mG, I, G, 5mU, 6 mA is generally higher for women in comparison to men. However this increase can be well explained by the effect of sex on creatinine clearance (Eq. 20) as one can expect an increase by a factor of 1.35 (1/0.742) due to sex-dependent excretion rate of creatinine. The value of nucleosides/creatinine excretion rates for MTA, is slightly lower, especially when corrected by sex-dependent creatinine excretion rate.

We also checked the fit of the developed model with and without incorporation of case/control effect to evaluate whether this covariate influences nucleosides/creatinine excretion rates. The DIC was found to be lower for model with case/control status in comparison to the model without this covariate (ΔDIC = 31), which indicates improvement of model accuracy.

### Probability of cancer development

The validation set was used to evaluate the model performance by calculating the probability of cancer for individuals with known nucleosides and creatinine concentration ratios, age and sex based on the model developed using the training set.

The accuracy of classification between patients and healthy individuals was summarized by area under the ROC, accuracy, sensitivity and specificity. The individual *a posteriori* prediction of disease occurrence along with 5th and 95th percentiles expressed via area under the ROC was 0.57 (0.5–0.67) with sensitivity and specificity of 0.59 (0.42–0.76) and 0.57 (0.45–0.7), respectively suggesting limited usefulness of nucleosides in predicting patients’ health status in this population.

The area under the ROC defines the relationship between the true positive rate (TPR, in other words a probability that a patient has a positive test result) and the false positive rate (FPR, a probability that a healthy individuals has a positive test result) from a binary response across all possible threshold values. The area under the ROC describes the probability that the outcome for a randomly drawn individuals from “diseased” group is higher than for a randomly drawn individuals from “healthy” group (Hossain et al. 2015).

We therefore conclude, that predictive performance of nucleosides and creatinine concentration modelled in a function of age and sex, characterized by *a posteriori* area under the ROC is far from being acceptable in terms of candidate disease indicators.

**Fig. 4:**
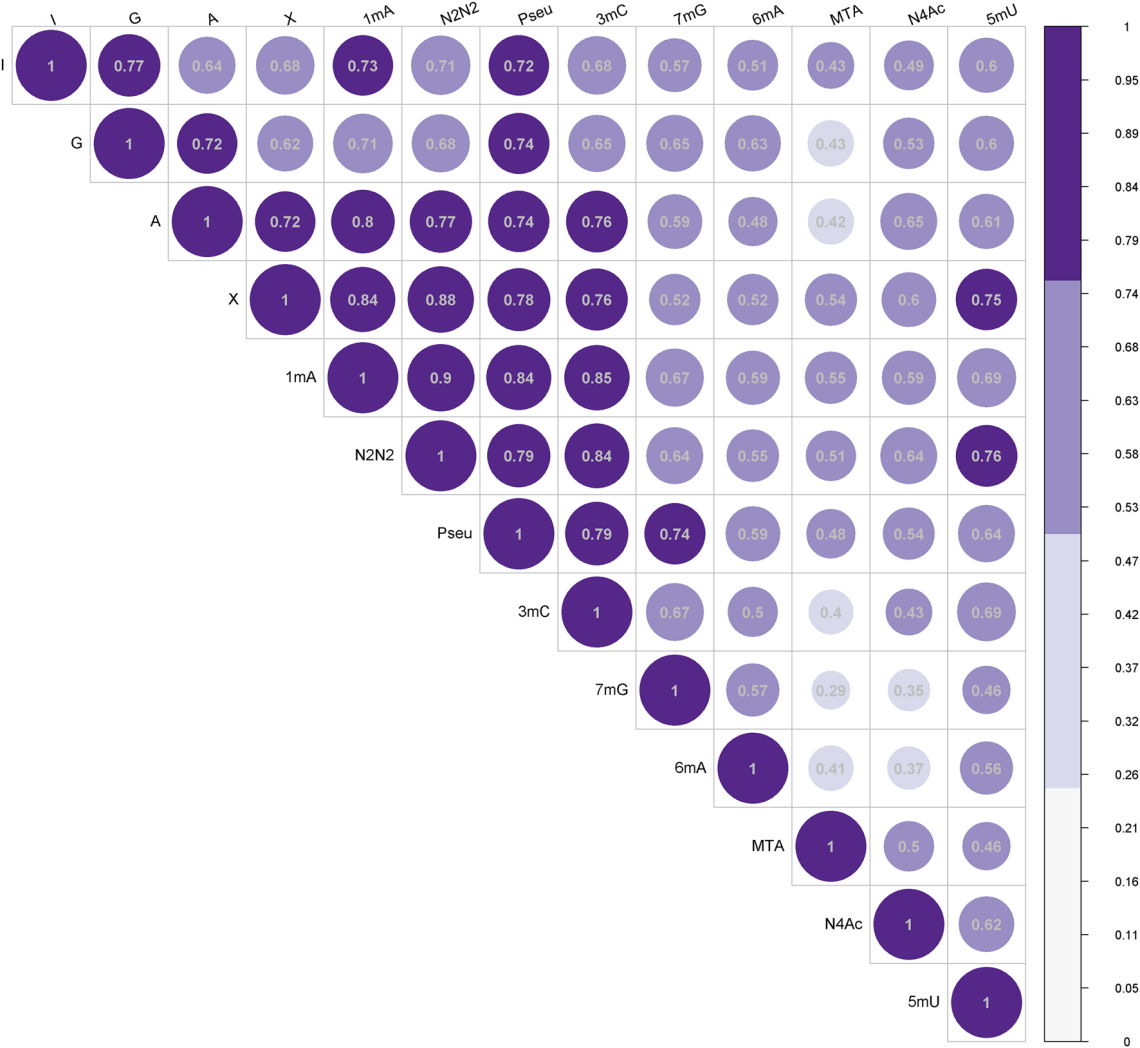
Correlation matrix demonstrating the degree of linear relationship beetween nucleosides. The variables are ordered according to the value of Pearson correlation coefficient. The lighter the color the lesser coefficient value between two variables

To evaluate how this conclusion is reflected in an individual person, we plotted the individual probability of cancer occurrence for randomly selected individuals from the validation set (Fig. 4). Based on that, we could make a rationale decision whether knowledge on nucleosides/creatinine excretion rate adds any information to the prior probability of cancer for an individual person. The concept of establishing an individual probability of disease occurrence lies within the foundation of decision theory and has long been recognized in neuroscience and cognitive research (Körding 2007). In this work, using Bayesian statistics within a decision framework, *a priori* we assumed equal probability of cancer occurrence (0–1) and no knowledge on health status of a patient or uncertainty associated with it. Following Bayesian concept, after seeing the data our probability for a particular patient is updated (posterior probability). The mean values of posterior distribution represent the most likely decision on the probability of cancer assuming the model and the available data. However, at this point it should be noted that prediction made for an individual patient can only be probabilistic due to uncertainty of a decision. In our opinion, the concept of using Bayesian statistics within a multilevel PK-driven modeling framework can be used in a decision framework and may have a practical usefulness in metabolomics regarding e.g. most beneficial choices made by physician for an individual patient in terms of diagnosis and treatment (Hozo et al. 2015).

### Biological inference on the effect of methylthioadenosine (MTA)

**Fig. 5:**
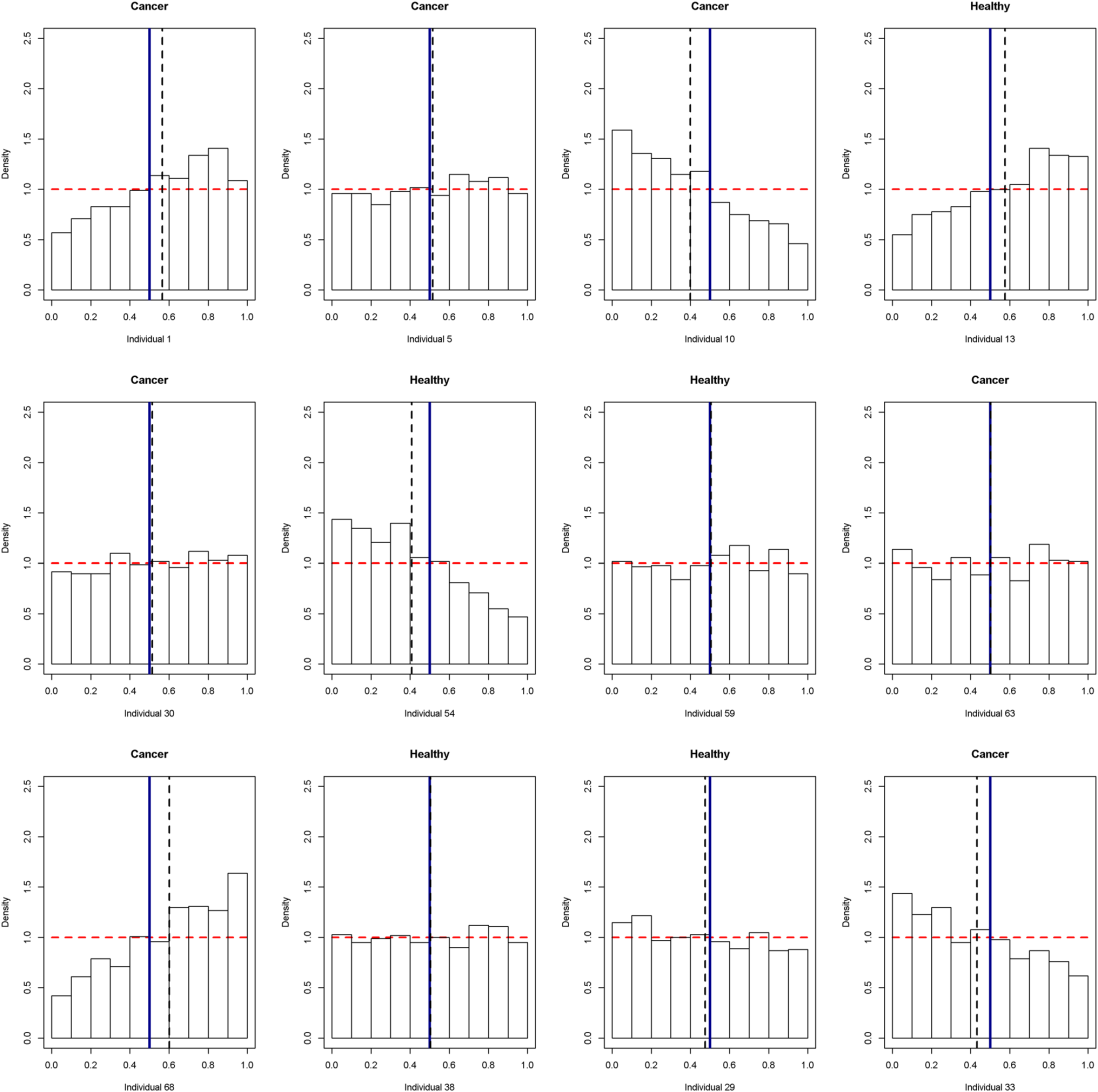
Individual distribution of prior and posterior probability of cancer estimated based on 13 nucleosides/creatinine excretion rates for 12 randomly selected individuals from the validation set. *Red dotted line* represents the prior assumed, histograms denote posterior probability distribution.* Vertical blue line* denotes the mean of posterior probability of cancer

The effect for MTA observed in the model assuming informative prior on age and sex and normally-distributed regularizing prior on case/control effect, is evident. Such increase in MTA concentration may not be significant from clinical viewpoint, however its physiological role deserves attention and has already been addressed in the literature. Therefore, we further evaluated the relationship between MTA and the remaining nucleosides, assessing the presence of linear relationship between them through the observed estimates of variance–covariance $\Lambda$. The MTA exhibited the lowest correlation coefficient in relation to the remaining nucleosides which may suggest different metabolic pathway for the remaining 12 nucleosides (Fig. 5).

MTA (5′-deoxy-5′-methylthioadenosine) is produced from S-(5′-deoxy-5′-adenosyl)-L-methionie during synthesis of polyamines, spermidine and spermine. MTA is a principal substrate for the methylthioadenosine phosphorylase (MTAP) enzyme, which claves the MTA into adenine and methylthioribose-1-phosphate (Nobori et al. 1996). Adenine is then salvaged to form AMP by the action of adenine phosphoribosyltransferase (Karikari et al. 2005), and this is the only known source of free cellular adenine. MTA has been shown to affect the expression of a variety of transcription factors and genes involved in growth and apoptosis (Andreu-Pérez et al. 2010).

The MTAP plays an important role in adenine and methylthio- moieties of MTA recycling back to the metabolites from which S-adenosyl-L-methionine is formed – ATP and methionine. All normal mammalian tissues contain MTAP which maintains cellular MTA at a very low level. Since cancer cells are commonly MTAP-deficient, MTA is not metabolized but accumulates in cells and /or is excreted (Stevens et al. 2008).

The mechanism behind MTAP deficiency lies in frequent homogenous co-deletion of MTAP gene with the CDKN2A gene, which is responsible for encoding p16 and p14ARF which are tumor suppressor proteins found in the chromosome 9p21 region. Lack of MTAP activity has been reported in approximately 40% of non-small cell lung, pancreatic, and biliary tract cancer, 70% of mesothelioma and glioblastoma, 35% of osteosarcoma, soft-tissue sarcoma, and T-cell acute lymphoblastic leukemia (Lubin et al. 2009). Molecular studies using hepatocellular carcinoma cells (HCC) demonstrated down-regulation of MTAP expression in human HCC cell lines and tissues as compared to primary human hepatocytes and non-tumorous tissue. LC–ESI–MS/MS revealed significantly higher intracellular and secreted MTA level as compared to non-tumorous liver tissue. Moreover in human HCC tissue, MTAP expression correlated inversely with MTA level. The MTAP down-regulation in HCC was proposed to be correlated with tumor staging and grading suggesting MTA as potential biomarker for tumor progression. However, still there is little information on the molecular mechanisms of how the reduction of MTAP expression and increased MTA levels affect cancer (Kirovski et al. 2011).

Biological background behind the increased excretion of modified and unmodified nucleosides is elusive. However, RNA turnover is known to be responsible for release of nucleosides thanks to the activity of ribonucleases and phosphatases. The same enzymes are capable to recycle ribonucleosides, such as cytidine, guanosine, uridine or adenosine for RNA rebuilding process in the commonly known salvage pathway. It’s been reported that modified nucleosides participate in tRNA discrimination, translation fidelity via codon–anticodon interaction, maintenance of reading frame, tRNA stability and quality control (Hopper 2013).

Moreover reduction, substitution or isomerization, to which RNA is subjected, produce modified nucleosides which cannot be recycled to rebuild RNA due to the lack of specific phosphorylases. However, these modifications are important in order to improve integrity, biological activity and efficiency of RNA at a biochemical level. Thus, any pathophysiological state or metabolic imbalance which affects RNA turnover or breakdown is assumed to influence the levels of excreted nucleosides (Hsu et al. 2013).

## Conclusions

The feature which distinguishes multilevel models from classical regression (used e.g. in metabolomics), relates to modeling the variation. Within this concept, we consider a multilevel model to be a regression model in which the parameters are provided a probability model. Accordingly, this second-level model has its own parameters (known as model hyperparameters) which are also estimated from data (Gelman et al. 2007). This idea of Bayesian multilevel modeling is based on conceptual knowledge and has already been applied in the field of natural and political science (McElreath 2014) and pharmacokinetics (Wakefield 1996; Wiczling 2016).

In this study we introduced the concept of Bayesian multilevel modeling to large-sample, unbalanced, sex and age-unadjusted targeted metabolomics data. Within this methodology, we provided physiologically-based PK model describing nucleoside-specific biology behind the urogenital tract cancer, related to covariates (age, sex, case/control status) and between-subject variability. The proposed methodology offers a new possibility for clinical decision making due to establishing an individual probability of disease occurrence. In this context, we think that Bayesian statistics within a decision framework can influence the field of biomarker studies in various aspects not only limited to metabolomics. Within biological context, we conclude limited usefulness of nucleosides to predict urogenital tract cancer in our population, however still information provided by nucleosides, should not be completely ignored. Especially the evidence of MTA association with cancer requires further studies to elucidate whether this effect is really caused by cancer or whether it is another factor associated with it.

To sum up, we conclude that dealing with both, large- and small-sample, unbalanced and unadjusted data sets forces the use of methods other than those based on classical hypothesis-significance-testing, which by definition were designed to investigate large and stable effects (however the methodology proposed can also be applied to balanced and adjusted datasets). In non-targeted metabolomics, measuring thousands of signals in a sample make the investigated effects highly context dependent, noisy with plenty of missing data points. In such case, multilevel Bayesian approach allows modeling concentrations below and above detection limit without the need to discard those data points prior modeling. Within the context of big data modeling, regularizing prior imposed on model parameters shrinks them toward zero simultaneously reducing their variance which further results in model overfitting reduction. The methodology proposed also allows to account for other sources of variability than between-subject and intra-subject variability, e.g. between-occasion, between-laboratory, between different analytical techniques. Simultaneously, we can estimate uncertainty around model parameters and predictions and reduce multiple testing problem.

Therefore, new methodologies for the analysis of metabolomics data should be adopted from other disciplines in order to make a proper inference from the data and provide a rational decisions in further clinical applications.

## Electronic supplementary material

Below is the link to the electronic supplementary material.

Supplementary material 1 (DOCX 16 KB)
