# Supplementary material for: Multilevel pharmacokinetics-driven modeling of metabolomics data
Source: Metabolomics. 2017 Feb 8;13(3):31. doi: 10.1007/s11306-017-1164-4 (PMC5306155; doi:10.1007/s11306-017-1164-4)
Supplement: Supplementary file 1 — Supplementary material 1 (DOCX 16 KB) [file 11306_2017_1164_MOESM1_ESM.docx]

**Multilevel pharmacokinetics-driven modeling of metabolomics data**

Emilia Daghir-Wojtkowiak, Paweł Wiczling*, Małgorzata Waszczuk-Jankowska, Roman Kaliszan, Michał Jan Markuszewski**

* wiczling@gumed.edu.pl, tel: +48 58 3491493, fax: +48 58 3491962

** markusz@gumed.edu.pl, tel: +48 58 3491493, fax: +48 58 3491962

Department of Biopharmaceutics and Pharmacodynamics, Medical University of Gdańsk, Al. Gen. Hallera 107, 80-416 Gdańsk, Poland, tel: +48 58 3491493, fax: +48 58 3491962

**# Load the data:**

myData.train = read.table("D:/Bayesian/Bayesian stat_nucleosides/codes/LASSO model_random div_train_test_all_cov/training.txt", header = T, sep="\t")

myData.val = read.table("D:/Bayesian/Bayesian stat_nucleosides/codes/LASSO model_random div_train_test_all_cov/validation.txt", header = T, sep="\t")

ty = myData.train$casecont

vy = myData.val$casecont

tX = myData.train[,c(6:18)]

vX = myData.val[,c(6:18)]

tage = myData.train[,4]

vage = myData.val[,4]

tgender = myData.train[,3]

vgender = myData.val[,3]

tNtotal = length(ty)

vNtotal = length(vy)

tPtotal = length(tX)

vPtotal = length(vX)

omega_inv_prior = diag(1,13)*0.05

logCobs = log(tX)

vlogCobs = log(vX)

**## center, scale concentrations**

df <- rbind(logCobs, vlogCobs); df <- as.matrix(df)

center_apply <- function(x) {

apply(x, 2, function(y) (y - mean(y)/sd(y)))

}# apply it

center_apply(df)

logCobs <- df[1:178, ];

vlogCobs <- df[179:248, ];

**## data list ##**

dataList = list( casecont = ty , casecontvalpred = vy, tNtotal = tNtotal , vNtotal = vNtotal , tPtotal = tPtotal, vPtotal = vPtotal, tX = as.matrix(tX), vX = as.matrix(vX), tage = tage, vage = vage, tgender = tgender, vgender = vgender, omega_inv_prior = omega_inv_prior, logCobs = as.matrix(logCobs), vlogCobs = as.matrix(vlogCobs))

**# Initial values #**

nChains = 3

for( i in 3: nChains) {

initsList = function(){

m.THETA = mvrnorm(n = 1, colMeans((logCobs)), Sigma = diag(1,13)*0.2)

m.FRA = mvrnorm(n = 1, rep(1,tPtotal), Sigma = diag(1,tPtotal)*0.1)

m.FRG = mvrnorm(n = 1, rep(1,tPtotal), Sigma = diag(1,tPtotal)*0.1)

m.FRC = mvrnorm(n = 1, rep(1,tPtotal), Sigma = diag(1,tPtotal)*0.1)

m.PPP = mvrnorm(n = 1, rep(1,70)*0.64, Sigma = diag(1,70)*0.005)

m.omega.inv = solve(diag(exp(2*rnorm(tPtotal,log(0.3),0.5))))

return( list( THETA = m.THETA ) )

return( list( FRA = m.FRA ) )

return( list( FRG = m.FRG ) )

return( list( FRC = m.FRC ) )

return( list( PPP = m.PPP) )

return( list( omega.inv = m.omega.inv ) ) }}

**# Run the chains:**

nChains = 3

nburnin = 6000

nAdaprSteps = 1000 # number of adaptive iterations to use at the start of the simulation

nUseSteps = 1000

nThinSteps = 6 # thinning interval

set.seed(1)

runjagsModel = run.jags(method = "parallel", model = "finalFF_model_22092016.txt", monitor= c("THETA","FRA","FRG","FRC","omega","logCobsPred","logCobsvPred", "casecontv", "PPP"), data=dataList , inits=initsList , n.chains=nChains ,adapt=nAdaprSteps, burnin = nburnin, sample = ceiling(nUseSteps/nChains), thin = nThinSteps);

**## Model development ####**

model {

for ( i in 1:tNtotal ) {

logCobs[i,1:13] ~ dmnorm(logthetaMean[i,1:13], omega.inv[1:13,1:13])

logCobsPred[i,1:13] ~ dmnorm(logthetaMean[i,1:13], omega.inv[1:13,1:13])

for ( j in 1:13 ) {

logthetaMean[i, j] <- THETA[j] + FRC[j]*casecont[i] + FRA[j]*log(tage[i]/50) + FRG[j]*tgender[i]}}

### cancer predictions on val set ###

for ( i in 1:vNtotal ) {

vlogCobs[i, 1:13] ~ dmnorm(vlogthetaMean[i, 1:13], omega.inv[1:13, 1:13])

for(j in 1:13){

vlogthetaMean[i, j] <- THETA[j] + FRC[j]*casecontv[i] + FRA[j]*log(vage[i]/50) + FRG[j]*vgender[i]}

}

#### nucleosides predictions for val set ###

for(i in 1:vNtotal ){

logCobsvPred[i, 1:13] ~ dmnorm(logthetaMeanvPred[i, 1:13], omega.inv[1:13, 1:13])

for(j in 1:13){

logthetaMeanvPred[i, j] <- THETA[j] + FRC[j] * casecontvalpred[i] + FRA[j] * log(vage[i]/50) + FRG[j] * vgender[i]}

}

# priors #

omega.inv[1:13, 1:13] ~ dwish(omega_inv_prior[1:13,1:13], 13)

omega[1:13, 1:13] <- inverse(omega.inv[1:13, 1:13])

for ( j in 1:13 ) {

THETA[j] ~ dnorm(0,0.0001)

FRC[j] ~ dnorm(0, tausigma1)

FRA[j] ~ dnorm(0.203 ,tausigma2)

FRG[j] ~ dnorm(0.293 ,tausigma3)

}

tausigma1 <- 1/(sigma1*sigma1)

sigma1~ dunif(0.001, 1000)

tausigma2 <- 1/(sigma2*sigma2)

sigma2~ dunif(0.001, 1000)

tausigma3 <- 1/(sigma3*sigma3)

sigma3~ dunif(0.001, 1000)

# priors val

for ( j in 1:vNtotal ) {

casecontv[j] ~ dbern(PPP[j])

}

for ( j in 1:vNtotal ) {

PPP[j] ~ dbeta( 1,1 )

}

}

codaSamples = as.mcmc.list( runjagsModel )

class(codaSamples)

samples1 <- as.array(codaSamples[[1]], drop=T, iters = T,chains = TRUE ) #

samples2 <- as.array(codaSamples[[2]], drop=T, iters = T, chains = TRUE ) #

samples3 <- as.array(codaSamples[[3]], drop=T, iters = T, chains = TRUE ) #

df <- rbind(samples1, samples2)

df2 <- rbind(df, samples3)

samples = df2; dim(samples);

samples <- as.data.frame(samples)

theta <- select(samples, starts_with("theta["));dim(theta) #1002/13

FRA <- select(samples, starts_with("FRA["));dim(FRA) #1002/13

FRG <- select(samples, starts_with("FRG["));dim(FRG) #1002/13

FRC <- select(samples, starts_with("FRC["));dim(FRC) #1002/13

logCobsPred <- select(samples, starts_with("logCobsPred["));dim(logCobsPred) #1002 2314

casecontv <- select(samples, starts_with("casecontv["));dim(casecontv) #1002 70

logCobsvPred <- select(samples, starts_with("logCobsvPred["));dim(logCobsvPred) #1002 910

ppp <- select(samples, starts_with("ppp")); dim(ppp) # 1002/70

omega <- select(samples, starts_with("omega["));dim(omega) #1002/169

**## Back to original scale ##**

df <- rbind(logCobs, vlogCobs); df <- as.matrix(df); dim(df) #orignical scale matrix

st.odch <- apply(df, 2, sd) #sd original scale

srednia <- apply(df, 2, mean) #mean original scale

theta1 <- as.matrix(theta) * st.odch + srednia

FRA1 <- as.matrix(FRA) * st.odch

FRG1 <- as.matrix(FRG) * st.odch

FRC1 <- as.matrix(FRC) * st.odch
